# Comfort, safety and quality of upper gastrointestinal endoscopy after 2 hours fasting: a randomized controlled trial

**DOI:** 10.1186/1471-230X-13-158

**Published:** 2013-11-09

**Authors:** Angélica Terezinha Koeppe, Marcio Lubini, Nilton Maiolini Bonadeo, Iran Moraes, Fernando Fornari

**Affiliations:** 1 Programa de Pós-Graduação: Ciências em Gastroenterologia e Hepatologia, Faculdade de Medicina, Universidade Federal do Rio Grande do Sul, Porto Alegre-RS, Brazil; 2 Faculdade de Medicina, Universidade de Passo Fundo, Passo Fundo-RS, Brazil; 3 Unidade de Endoscopia Digestiva, Hospital São Vicente de Paulo (HSVP), Passo Fundo-RS, Brazil; 4 School of Medicine, Programa de Pós-Graduação: Ciências em Gastroenterologia e Hepatologia, Universidade Federal do Rio Grande do Sul, Rua Ramiro Barcelos, 2400, 2° andar, 90035-001, Porto Alegre-RS, Brazil

**Keywords:** Clear liquids, Short fasting, Comfort, Upper GI endoscopy

## Abstract

**Background:**

Upper gastrointestinal endoscopy has been performed after fasting 8 or more hours, which can be harmful to the patients. We assessed comfort, safety and quality of endoscopy under moderate sedation after 2 hours fasting for clear liquids.

**Methods:**

In this clinical trial, patients referred for elective endoscopy were randomly assigned to a fasting period of 8 hours (F8) or a shorter fasting (F2), in which 200 ml of clear liquids were ingested 2 hours before the procedure. Endoscopists blinded to patients fasting status carried out the endoscopies. Comfort was rated by the patients, whereas safety and quality were determined by the endoscopists.

**Results:**

Ninety-eight patients were studied (aging 48.5 ± 16.5 years, 60% women): 50 patients (51%) in F2 and 48 in F8. Comfort was higher in F2 than F8 in regard to anxiety (8% vs. 25%; P = 0.029), general discomfort (18% vs. 42%; P = 0.010), hunger (44% vs. 67%; P = 0.024), and weakness (22% vs. 42%; P = 0.034). Regurgitation of gastric contents into the esophagus after endoscopic intubation did not differ between F2 and F8 (26% vs. 19%; P = 0.471). There was no case of pulmonary aspiration. Gastric mucosal visibility was normal in most patients either in F2 or F8 (96% vs. 98%; P = 0.999).

**Conclusions:**

Elective upper GI endoscopy after 2 hours fasting for clear liquids was more comfortable and equally safe compared to conventional fasting. This preparation might be cautiously applied for patients in regular clinical conditions referred for elective endoscopy.

**Trial registration:**

SAMMPRIS ClinicalTrial.gov number, NCT01492296

## Background

Upper gastrointestinal (GI) endoscopy is a procedure widely used in the medical practice. The guiding principle of this procedure must be patient comfort and safety. Thus, endoscopy is conventionally performed under moderate sedation and after a fasting period of at least 6 hours [1,2]. In the practice, the routine prescription of *non per os* from midnight usually extends the fasting period for 8 or more hours [3]. A long fasting period has been justified by the need of an empty stomach during endoscopy, allowing proper visualization of the upper GI mucosa. In addition, the risk of pulmonary aspiration is minimized in the absence of gastric residua [4,5]. However, a prolonged fasting is often associated with patient’s complaints, such as weakness, thirst, anxiety and hunger [6-8]. Moreover, a long fasting period may increase the risk of complications, particularly in elderly patients, including nutritional, fluid and glycemic imbalance [9,10].

In the last decades, a shorter fasting has been tested with success before surgical procedures requiring general anesthesia [11,12]. Evidence from studies including metanalyzes has forced medical societies to change their guidelines, proposing a reduction of preoperative fasting [13-16]. The ingestion of clear fluids containing carbohydrates few hours before elective procedures has been shown to be safe, resulting in similar volumes of gastric residua without pH changes in comparison to more prolonged fasting periods [17-19]. For upper GI endoscopy, at least two studies have looked to this question, showing good visibility and improved comfort with the ingestion of water just before endoscopy [20,21]. However, further evidence is still needed in order to change the paradigm of prolonged fasting before upper GI endoscopy. Despite an acceptable safety level demonstrated in these studies, it is unknown whether a 2 hours fasting is suitable for upper GI endoscopy. The ingestion of a clear solution containing electrolytes and nutritional components, of easy gastric emptying, might decrease endoscopy-related discomfort and fasting-related risks.

We hypothesize that drinking clear liquids 2 hours before upper gastrointestinal endoscopy makes the procedure more comfortable than that performed after a conventional longer fasting, with no substantial compromising in safety and quality. We therefore designed a randomized trial to test this hypothesis.

## Methods

### Patients

In this parallel-group, single-blind, randomized controlled trial, patients who were referred for elective upper endoscopy at our Institution between March and November 2011 were considered eligible for the study, either outpatients or hospitalized. The inclusion criteria were the following: 1. Age ≥ 18 years; 2. Agreement in participate, both from the patient and its physician. Patients were excluded in the presence of the following: 1. Nasoenteral feeding tube; 2. Unstable clinical condition; 3. Morbid obesity; 4. Active GI bleeding; and 5. Gastroesophageal surgery. Patients were randomized to drink clear liquids 2 hours before endoscopy (group F2) or to perform a conventional 6–8 h fasting (group F8).

The study was conducted in accordance with the Helsinki Declaration and was approved by the Ethical Committee of Universidade de Passo Fundo (CAAE n° 0217.0.398.000-10, n° 378/2010). All patients and their respective physicians signed informed consent before entry in the study. Fresenius Kabi played no role in the design of the study, in data collection or analysis, or in manuscript preparation.

### Endoscopy

Upper endoscopy was performed under moderate sedation either with endovenous midazolan (0.03-0.05 mg/kg) or a combination of endovenous midazolan and fentanyl (1 μg/kg). Patients were categorized according to ASA physical status classification before endoscopy. Topical pharyngeal anesthesia with lidocaine spray was applied to all patients before sedation. The procedures were carried out using videoendoscope Olympus CF-130 (Olympus, Japan) and Fujinon EG 2200 (Fujinon, Japan). Four experienced endoscopists (ML, NMB, IMJ, FF) were trained to be familiar with the study protocol and were oriented to perform the procedures blinded to the patients fasting status. After the end of endoscopy the examiners replied to a structured interview in order to assess safety and quality of each procedure.

### Conventional and short fasting

Patients were asked to eat their usual diet containing food and liquids before entering in the fasting protocols. Those assigned to the conventional fasting period were instructed to remain at least 6 hours in fasting conditions. However, considering that most patients had a prescription of *non per os* after midnight, we decided to standardize these patients as doing an 8 hours fasting. Patients assigned to the short fasting were instructed to avoid ingestion of food and liquids for 6 hours and then were asked to drink 200 ml of clear liquids (ProvideXtra Drink®, Fresenius Kabi, São Paulo, Brazil) 2 hours before the endoscopy. This solution is a nutritional complement constituted of hyperchaloric (1.5 Kcal/ml) clear liquid containing carbohydrates (maltodextrin and sacarose), pea-derived proteins (8 g/200 ml) and sodium (194 mg/200 ml). It is free of lipids and fibers.

### Study protocol

Patients with request of endoscopy for one of our examiners (ML, NMB, IMJ, FF) were identified between March and November of 2011, either hospitalized or outpatients. A nurse (ATK) enrolled the participants. After agreement to participate, patients were asked to remain in fasting conditions between 6 and 8 hours before randomization. Two hours prior to endoscopy, patients were randomly assigned to drink 200 ml of clear liquids or to remain fasting. The draw was performed by a technician who did not participate of the other study steps, using a luck dice (even = F8; odd = F2). This technician supervised the intake of clear liquids from F2 patients. The participants Patients were asked to avoid commentaries about their fasting status in the endoscopy room and during post-procedure interview. A nurse (ATK) blinded to patients fasting status followed all the procedures, taking note of clinical data, indication of endoscopy, patient weight and height, sedatives and procedure length. Immediately after the end of the endoscopies, the examiners replied to a structured interview designed to assess safety and quality. After recovery from sedation, patients were interviewed by the same nurse to assess comfort.

### Study outcomes

Patient comfort was elected as the primary outcome, given that sample size was calculated according to effect estimation on this parameter. Endoscopy-related safety and quality were considered as secondary outcomes. Structured interviews were carried out to assess comfort according to patients, and safety and quality according to endoscopists. The questionnaire applied to the patients was composed of 6 questions assessing fasting-related symptoms. The first question was ‘did you feel hunger during the fasting period?’. The 5 subsequent questions had the word ‘hunger’ changed to thirst, weakness, anxiety, nausea and discomfort. The questionnaire applied to the endoscopists included 5 questions regarding safety and 2 questions approaching quality (Table 1). Questions about safety generated categorical answers (yes or not), whereas quality was assessed by a question with categorical answer addressing visibility (normal/compromised) and a question with quantitative reply addressing general quality, ranging between 1 and 10 (best), as a Likert scale.

**Table 1:** Structured interview designed to assess safety and quality of endoscopy

	** Safety questions (answers: yes or not)**
1.	Did you notice the occurrence of nausea or vomit before the endoscopy?
2.	There was regurgitation of gastric content after endoscopic intubation?
3.	Did you observe stasis of liquid in the gastric lumen?
4.	Did you find stasis of food in the gastric lumen?
5.	Was the risk of tracheal aspiration increased?
	** Quality questions**
6.	Was the visibility of the gastric mucosa compromised? (yes or not)
7.	Overall, which rate would you give in relation to quality? (1 to 10 [best])

Answers were indicated by the endoscopists following the procedure.

### Sample size and statistical analysis

We calculated that 45 patients were needed in each group in order to detect a difference of 30% in comfort in favor of patients who fasted 2 hours (two-sided test; alpha level, 0.05; beta level, 0.80). All randomized patients completed the study.

Data are presented as mean ± SD or frequencies and percentages. Quantitative data were analyzed with Student *t* test, whereas qualitative data were analyzed using Fisher exact test or chi-square test. The analyses were performed with GraphPad Prism 4 (GraphPad software, Inc., San Diego, CA, USA). A P value of < 0.05 was assumed as indicative of statistical significance.

## Results

### Patients

**Figure 1:**
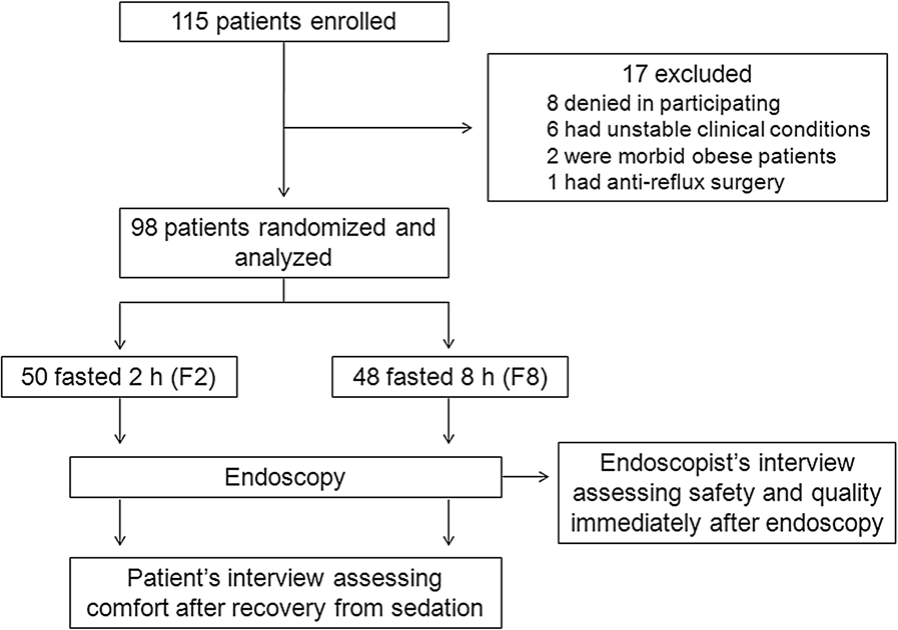
Enrollment, randomization and follow-up of participants.

A total of 115 patients were interviewed in the enrollment phase of the study (Figure 1). Of these, 17 patients (14.8%) were excluded due to the following reasons: 8 denied in participating, 6 were in unstable clinical conditions, 2 had morbid obesity, and one patient had anti-reflux surgery. After randomization, 98 patients composed the study population: 50 (51%) fasted for 2 hours (F2), while 48 patients fasted for 8 hours (F8). All enrolled patients completed the study protocol and were included in the analysis. The four endoscopists were interviewed immediately after each endoscopy, while the patients were interviewed after recovery from sedation, which occurred on average 4 hours after the procedure. Basal characteristics of the patients did not differ statistically between F2 and F8 (Table 2). As a group, patients aged around 50 years old, with a BMI around 25 kg/m^2^. The proportion of women/men was 6/4, and approximately two-thirds were hospitalized. Indications for endoscopy were investigation of gastroesophageal reflux disease (GERD) or dyspepsia (~80%), followed by others including atypical GERD symptoms, non-achalasic dysphagia, biliar disease, compensated liver disease and non-advanced gastrointestinal neoplasia. Diabetes mellitus was present in approximately 10% of the patients. ASA I was found in half of patients in both F2 and F8 groups, while the remainder patients were ASA II.

**Table 2:** Baseline characteristics of the patients

	**F2 (n = 50)**	F8 (n = 48)*
Age in years, mean ± SD	49.1 ± 16.9	47.8 ± 16.3
Women, n (%)	29 (58)	30 (62)
BMI, mean ± SD	24.9 ± 4.6	26.1 ± 5.1
Inpatient, n (%)	30 (60)	32 (67)
Indication, n (%)		
GERD	20 (40)	18 (38)
Dyspepsia	20 (40)	18 (38)
Others**	10 (20)	12 (24)
Diabetes mellitus, n (%)	5 (10)	4 (8)
ASA classification, n (%)		
I	25 (50)	24 (50)
II	25 (50)	24 (50)

F2 and F8 were patients who fasted 2 and 8 hours respectively.

*P-value > 0.05 in all comparisons between groups. **Atypical GERD symptoms, non-achalasic dysphagia, biliar disease, compensated liver disease and non-advanced gastrointestinal neoplasia.

### Comfort

**Figure 2:**
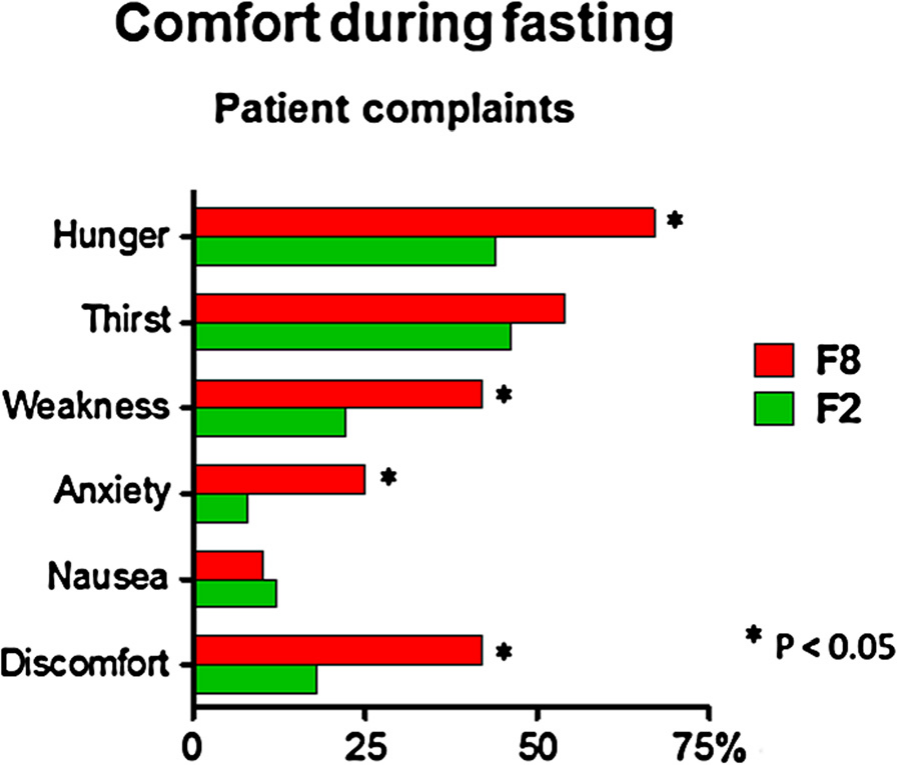
Comfort of patients who fasted 8 (F8) or 2 (F2) hours.

In comparison to F8 (Figure 2), less patients of F2 complained of hunger (44% vs. 67%; P = 0.024), weakness (22% vs. 42%; P = 0.034), anxiety (12% vs. 33%; P = 0.029) and fasting-related discomfort (18% vs. 42%; P = 0.010). The report of thirst (46% vs. 54%; P = 0.419) and nausea (12% vs. 10%; P = 0.999) was similar between F2 and F8. A separate analysis of hospitalized and outpatients revealed that the occurrence of fasting-related discomfort did not differ in both F2 (hospitalized 30% vs. outpatients 10%; P = 0.130) and F8 groups (47% vs. 31%; P = 0.300). Fasting-related discomfort was also equally reported by ASA I and ASA II patients in both F2 (24% vs. 12%; P = 0.463) and F8 groups (37% vs. 46%; P = 0.558).

### Safety

**Figure 3:**
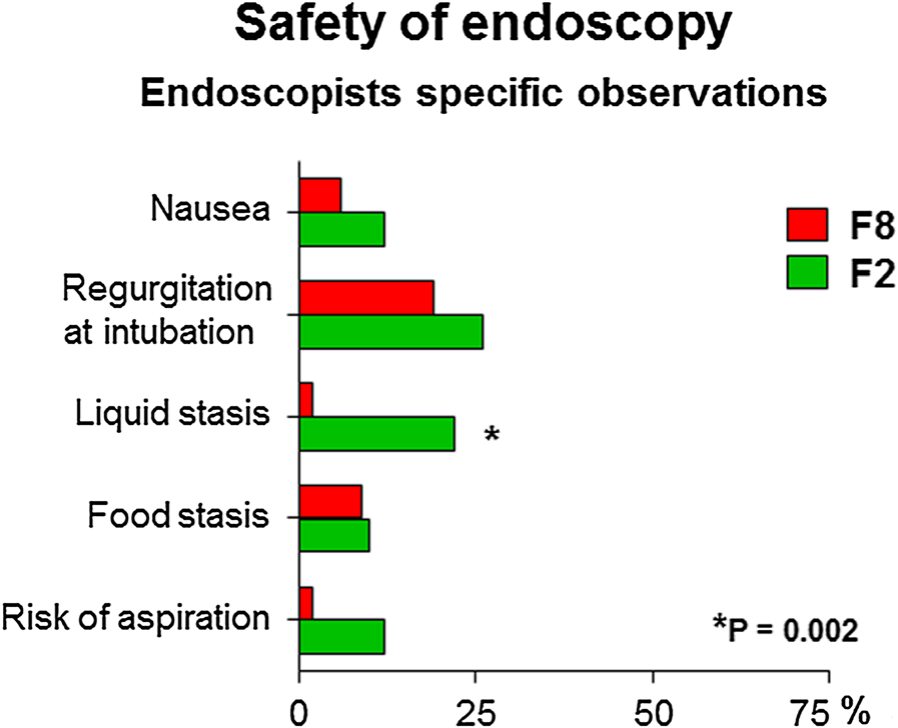
Safety of endoscopy in patients who fasted 8 (F8) or 2 (F2) hours.

**Figure 4:**
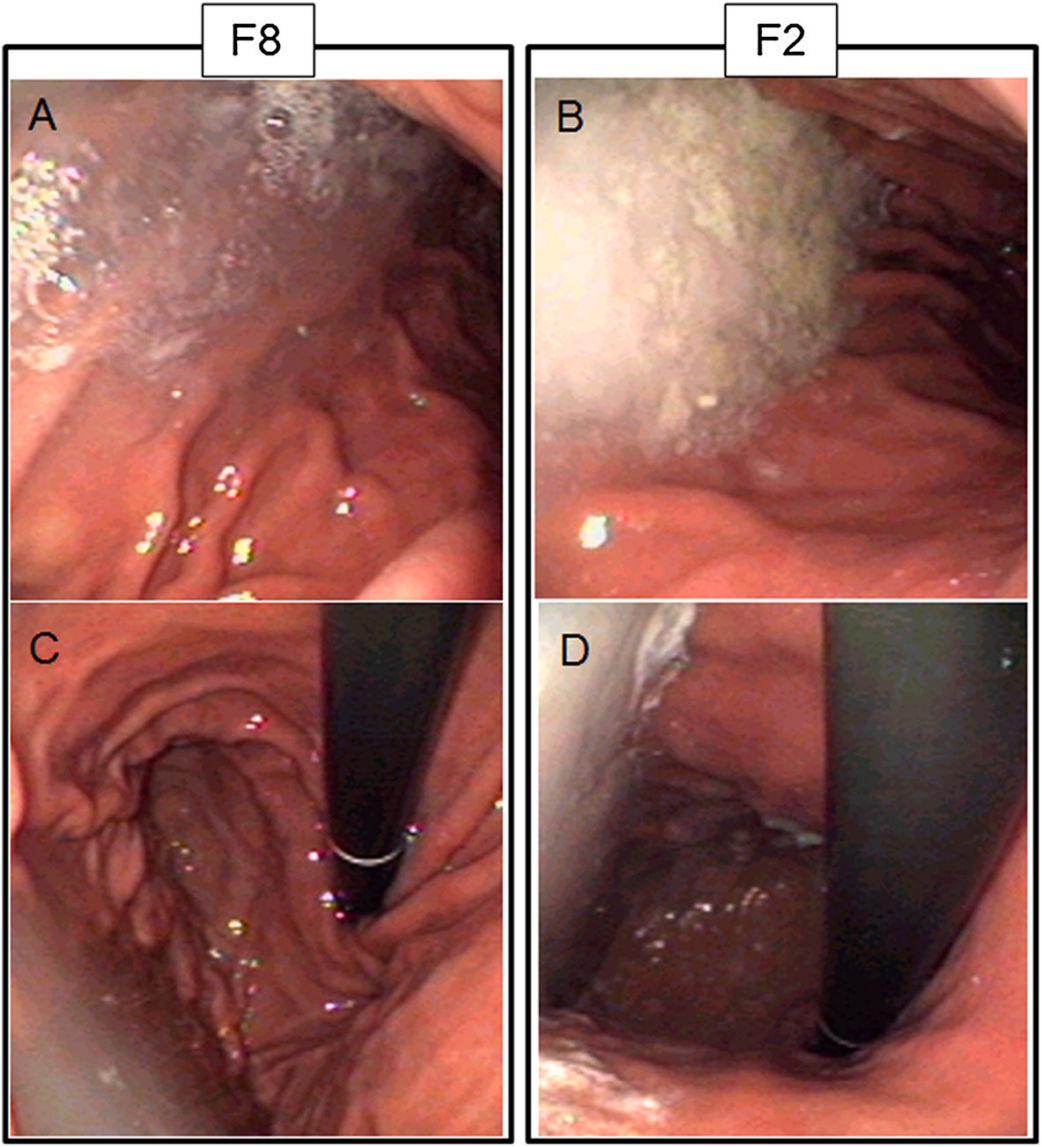
**Endoscopic view of the gastric lumen of patients who fasted 8 (F8) or 2 (F2) hours.** Note that the amount of liquid is subjectively higher in F2 patient. (**A** and **B**: frontal view of the gastric corpus in F8 and F2, respectively; **C** and **D**: retroview of the fundus in F8 and F2, respectively).

According to subjective evaluation of endoscopists (Figure 3), a higher proportion of F2 patients (n = 11, 22%) showed an increased amount of liquid in the gastric fundus liquid stasis in the stomach compared to F8 patients (n = 1, 2%; P = 0.002) (22% vs. 2%; P = 0.002). Examples are presented in Figure 4. However, no case of pulmonary aspiration was observed in either group. The other parameters did not differ statistically between F2 and F8, including nausea immediately before endoscopy, regurgitation after endoscopic intubation, food stasis in the stomach, and risk of aspiration (data not shown). Length of endoscopy did not differ between F2 and F8 (4 min 13 sec ± 1 min 14 sec vs. 4 min 3 sec ± 49 sec; P = 0.765). The amount of midazolan used for sedation was also similar between F2 and F8 (6.3 ± 2.1 mg vs. 6.8 ± 2.7 mg; P = 0.317). Among the five patients with diabetes mellitus who fasted 2 hours, there was no case of regurgitation of gastric contents into the esophagus after endoscopic intubation or increased risk of aspiration according to endoscopists judgment.

### Quality

**Figure 5:**
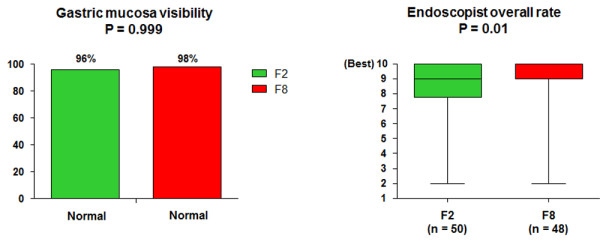
Quality of endoscopy in patients who fasted 8 (F8) or 2 (F2) hours.

The quantitative question of quality resulted in a high and equal score in both groups (Figure 5), but was statistically different between F8 and F2 patients [median (IQR25%-75%): 9 (9–10) vs. 9 (7.7-10); P = 0.010]. Quality was also higher in hospitalized patients than in outpatients who fasted 8 hours [median (IQR25%-75%): 10 (9–10) vs. 9 (7–9); P = 0.002]. Such score was numerically higher in hospitalized patients who fasted 2 hours compared to outpatients [9 (8–10) vs. 8 (7–9); P = 0.166], but without statistical significance. The comparison of ASA I and ASA II patients revealed no difference in terms of quality, either in F2 (P = 0.166) or F8 groups (P = 0.125). Visibility of the gastric mucosa was classified as normal in most cases, either in F2 or F8 (96% vs. 98%; P = 0.999).

## Discussion

The aim of this study was to assess comfort, safety and quality of endoscopy under moderate sedation after 2 hours fasting for clear liquids. For this purpose, we randomly assigned patients who were referred for elective endoscopy to perform the procedure either after a conventional fasting period of 8 hours or after 2 hours fasting for a carbohydrate beverage with rapid gastric emptying [22,23]. Comfort was rated by patients after recovery from sedation, whereas safety and quality were determined by endoscopists, blinded to patients fasting status.

In this randomized and controlled trial, we found that upper GI endoscopy after 2 hours fasting for clear liquids was more comfortable than the conventional procedure, particularly in terms of hunger, weakness, anxiety and general discomfort. We also found that a shorter fasting period did not compromise endoscopy safety. Although overall quality was slightly inferior after a shorter fasting, gastric mucosa visibility was not compromised in patients who drank clear liquids 2 hours before.

To the best of our knowledge, this is the first study evaluating endoscopy-related comfort after ingestion of a carbohydrate solution. Comfort was superior in patients who ingested clear liquids 2 hours before the endoscopy. In this group, fewer patients complained of hunger, weakness, anxiety and general discomfort. However, no significant advantage was observed in terms of thirst with a shorter fasting. This could be explained by a sweet taste of the ingested fluid. We have to mention other potential benefits of drinking a carbohydrate beverage before endoscopy, such as attenuation of both insulin resistance and organic response to stress [24]. The practice of a prolonged fasting before elective procedures such as endoscopies and surgeries has been recently questioned by authorities and societies [7,13]. Nevertheless, the prescription of ‘*non per os’* after midnight continues to be widely employed, despite the convincible evidence indicating that a shorter fasting is more comfortable and equally safe [14].

An empty stomach is required to ensure safety of procedures such as upper GI endoscopy [1]. Studies on gastric physiology have demonstrated that clear liquids are quickly emptied from the stomach, especially if free of lipids [25]. For instance, the emptying of water begins immediately after ingestion, with a half-emptying time of approximately 20 min. Despite the well-known rapidity of gastric emptying for clear liquids, the presence of residua in the gastric lumen has traditionally been assumed as a concern for pulmonary aspiration during interventions which involve pharyngeal manipulation [13]. In the present study, the ingestion of 200 ml of clear liquids 2 hours before upper GI endoscopy did not compromise procedure safety, according to evaluation of experienced endoscopists in a blinded fashion setting. Regurgitation of gastric contents into the esophagus following endoscopic intubation was equally observed in a minority of patients, regardless of fasting 2 or 8 hours. Additionally, neither procedure length nor midazolam dose was modified with a shorter fasting period. We also found that ASA status, including categories I and II, had no influence in study outcomes.

Prior reports have demonstrated that the ingestion of clear liquids until 2 hours before general anesthesia does not result in increased amount of gastric residua or changes in gastric pH [26,27]. Others have indicated that a short fasting for clear liquids may be accompanied by a lower amount of liquid in the gastric lumen in comparison to the conventional, longer fasting [17,19,28]. In a recent metanalysis, healthy adult participants given a drink of water preoperatively were found to have a significantly lower volume of gastric contents than the group that followed a standard fasting regimen. However, this difference was modest and clinically insignificant [14]. It has also been demonstrated that the ingestion of non-caloric solutions such as pronase diluted in 100 ml of water immediately before endoscopy did not compromise endoscopy safety [21]. Here we observed that patients who fasted 2 hours presented a higher amount of liquid in the gastric lumen than patients who fasted 8 hours, despite of subjective evaluation. Methodological differences could explain such controversies, pointing to the need of further studies with objective measurement of gastric residua during endoscopy.

In our study, endoscopy quality according to endoscopists judgment was considered high in both groups (median 9). Although F8 rate was statistically superior than F2, such difference may be irrelevant from the clinical point of view. In agreement, De Silva et al. have suggested that water ingestion 1 hour prior to endoscopy gives good endoscopic visibility [20]. Indeed, gastric mucosa visibility was normal in the majority of our patients and did not differ between F2 and F8 according to our endoscopists. Another recent study have shown that the administration of carbohydrate-rich drink until 2 and 4 hours before general anesthesia did not change quality of endoscopy [29]. Interestingly, endoscopy quality was higher in hospitalized patients than in outpatients, particularly in those who fasted 8 hours. This could reflect a more strict preparation carried out in the hospital setting.

We acknowledge limitations. Apart from a single-center study, we did not report data on oxygen desaturation, and cardiovascular parameters during endoscopies and gastric juice pH, as described in other studies [20,30]. We also did not objectively measure gastric residua, making questionable the finding of increased stasis of liquid in F2 patients. Despite the lack of complications in patients with diabetes, a limited number of participants precluded conclusions concerning the safety of a shorter fasting in patients with such condition. Finally, one may question the use of a lucky dice for patient randomization. However, we believe that bias chance was low given that baseline characteristics of F2 and F8 were similar and that follow up was short enough to ensure blinding.

## Conclusions

We assessed comfort, safety and quality of upper GI endoscopy with moderate sedation after fasting 2 hours for clear liquids. Our data suggest that this preparation is more comfortable and equally safe as compared to the traditional procedure. Although quality was slightly decreased despite normal gastric mucosa visibility, a shorter fasting period might be cautiously applied for patients in regular clinical conditions referred for elective upper GI endoscopy. However, studies with larger number of participants are advisable to confirm safety of this procedure.

## Abbreviations

F2: Fasting period of 2 hours; F8: Fasting period of 8 hours; GI: Gastrointestinal; ASA: American Society of Anesthesiologists; GERD: Gastroesophageal reflux disease; HSVP: Hospital São Vicente de Paulo.

## Competing interests

The authors declare that they have no competing interests. HSVP provided technical support for accomplishment of endoscopies.

## Authors’ contributions

KAT and FF conceptualized and designed the study, examined the patients, organized and analyzed the data and wrote the paper; LM, BNM and MJrI examined the patients and approved the final version of the article. All authors read and approved the final form of the manuscript.

## Pre-publication history

The pre-publication history for this paper can be accessed here:

http://www.biomedcentral.com/1471-230X/13/158/prepub
